# Enhancing Mixed Reality Simulation Training Technology With Real-Time Performance Visualization: Mixed Methods Study With Medical First Responders

**DOI:** 10.2196/57655

**Published:** 2024-12-24

**Authors:** Olivia Zechner, Helmut Schrom-Feiertag, Rafael Wespi, Daniele Pretolesi, Quynh Nguyen, Manfred Tscheligi

**Affiliations:** 1 Center for Technology Experience, Austrian Institute of Technology, Giefinggasse 4, Vienna, 1221, Austria, 43 505504506; 2 Department for Artificial Intelligence and Human Interfaces, University of Salzburg, Salzburg, Austria; 3 Department of Emergency Medicine, Inselspital, University of Bern, Bern, Switzerland

**Keywords:** mixed reality, immersive technologies, simulation training, simulation, paramedic, medical first responders, human performance, stress, stress monitoring, human-centered design

## Abstract

**Background:**

Mixed reality (MR) simulation training is emerging in paramedical education as a way to practice responding to stress-intensive scenarios like mass casualty incidents in a safe and controlled environment. Current training platforms, however, lack real-time stress and human performance monitoring tools.

**Objective:**

The study aims to enhance MR training for medical first responders through real-time evaluation of performance and stress levels, leveraging biosignal monitoring and advanced analytics to allow instructors to tailor feedback and maintain optimal challenge and safety levels.

**Methods:**

The study includes a structured, multiphase approach including initial requirement gathering (structured interviews and cocreation workshops), an online design survey, iterative prototype development, and a field trial (including training observations and interviews). Data were collected from 5 end user consortium members across Europe. Quantitative data from checklists were analyzed using frequencies and percentages to understand feature usage and event occurrences. Qualitative data from semistructured interviews and cocreation workshops were transcribed, coded, and subjected to thematic analysis to identify patterns and insights into the usability and effectiveness of the enhanced features in the MR training.

**Results:**

The study identified a number of requirements that medical first responders have for an MR training system, including requirements not included in currently available solutions. A total of 80 performance metrics were initially identified and refined to a set of 54 metrics, which were categorized into key performance indicator groups such as scene safety, triage performance, and communication. Requirements for smart wearables to monitor stress levels are provided and highlight the importance of a user-centered design process to provide users with effective tools that fit their needs. Stress visualization preferences are described in the form of a dashboard as well as in virtual environments surrounding the avatar. Using an iterative design process and user feedback, a training system was developed, integrating real-time performance tracking and stress monitoring. The field trial provided insights into the practical use of these features during a real training exercise, showed interaction preferences between trainer and trainees, and highlighted further improvement opportunities.

**Conclusions:**

This research enhances MR training for paramedics by integrating real-time performance metrics and stress indicators based on a human-centered design approach that aligns with end user needs, thereby laying the foundation for developing more effective and immersive training solutions for high-stress professions.

## Introduction

### Background

Medical first responders (MFRs) play a critical role in saving lives and providing emergency care in challenging and stressful situations. The number of mass casualty incidents (MCIs) has significantly increased in recent years [1]. However, providing effective training for MFRs can be a difficult task due to the complexity and unpredictability of emergency scenarios. Training methods for first responders are still analog, with minimal presence of digital and immersive technologies. For instance, the majority of training continues to adhere to conventional exposition-style instruction based on theories and examples presented in books and presentations [2]. This type of training can improve learning but primarily targets cognitive memory rather than physical/muscle memory [3]. Since the majority of training and operations in the first responder industry adhere to repetitive instructional designs and protocols, physical memory is considered to be of utmost significance [4].

Extended reality (XR) simulation training provides the opportunity to train a multitude of scenarios that capture all training possibilities found within mass casualty or natural disaster events without the extensive resources needed for real-life simulation training [5]. MFRs are offered new training methods based on learning management systems and XR training as a result of recent advancements in the field of immersive technologies. Due to the service’s portability and accessibility, XR learning management systems have been found to improve student retention and engagement [6].

With an increased interest in XR simulation training, which includes virtual reality (VR), augmented reality, and mixed reality (MR) [7], the need emerges for novel training interfaces that offer real-time feedback and support for both trainees and instructors. The possibility to digitalize and automatically track training performance metrics as well as psychophysiological metrics such as stress and cognitive workload bring considerable advantages in terms of training quality as well as efficiency. However, incorporating these key performance indicators (KPIs) into XR training platforms remains a relatively unexplored field in MFR training.

### Objectives

This study aims to enhance MR training solutions for MFRs by addressing the specific needs of end users and incorporating their iterative feedback throughout the development process. By integrating real-time performance evaluation and stress level monitoring through advanced KPIs and biosignal analysis, this research seeks to optimize training outcomes. The objective is to enable instructors to deliver feedback based on objective data, ensuring a balanced approach that maximizes both the challenge and safety of the training experience. Through these innovations, the study aims to fill existing gaps in MR training, ultimately improving preparedness and response effectiveness in emergency scenarios.

The research addressed the following research questions:

Research question 1. What are the key performance metrics in MR training that enable trainers to effectively engage with and mentor MFR trainees?

Research question 2. How can stress level indicators be integrated into an MR training platform tailored for MFRs?

### Related Work

The application of XR technologies in emergency response training has gained substantial interest in recent years [8-11]. These technologies offer a safe and controlled environment for rehearsing emergency scenarios without the associated risks [12,13]. Studies have demonstrated that XR training can achieve performance outcomes on par with, if not superior to, traditional training methods, making it a practical solution for complex simulations like MCIs and triage [14-16]. Unlike traditional large-scale exercises, which are resource-intensive and logistically demanding, XR training has been shown to be more cost-effective, though the initial investment in hardware and software remains a consideration [17,18].

Although XR has the potential to transform health care training [19], the research on its application for MFRs in MCIs is still in its early stages, revealing both promising results and notable limitations. Positive findings indicate that XR simulations provide an immersive and engaging experience [20], with participants reporting a heightened sense of presence and increased confidence in their abilities [21]. Studies comparing XR training to live simulations have found comparable efficacy in knowledge acquisition, decision-making skills, and user satisfaction, with no significant difference in the accuracy of triage decisions between paramedic students trained using XR and those who underwent live simulations [14,22]. Furthermore, XR offers a cost-effective and safe environment for learning, enabling repeated practice without real-world consequences [17,23].

Despite these benefits, a critical gap in the literature is the integration of real-time performance monitoring within MR training environments. Current studies primarily focus on the immersive qualities of MR and its ability to mimic real-world scenarios, often neglecting the necessity for immediate performance feedback. Effective training requires accurate and prompt evaluation of trainees’ actions, essential for ensuring that the skills acquired in virtual environments transfer effectively to real-world emergencies. Kirkpatrick’s model [24] suggests that evaluating training effectiveness comprehensively requires assessing reaction, learning, behavior, and results. Identifying specific, measurable, action-oriented, relevant, and timely KPIs is crucial [25,26]. Baetzner et al [27] found that most MFR training studies rely on an overall performance score, task completion time, and decision accuracy as external measures. However, self-reported metrics dominate these studies, underscoring the need for more objective evaluation methods since traditional measures are prone to different biases [28]. As training designs evolve and incorporate new technologies like XR, it is critical to maintain rigorous evaluation methodologies to ensure the transfer of training to real-world applications [29]. Digitalization of measurements in VR solutions presents an exciting opportunity to integrate more nuanced and continuous evaluation metrics, which can further enhance the effectiveness of MFR training [30].

Given the high-pressure nature of MCIs, it is imperative to incorporate the assessment of cognitive factors such as stress in simulation training. Evaluating these factors provides a holistic understanding of a trainee’s capacity to perform under stress, ensuring that they are not only technically proficient but also mentally resilient when faced with real-world emergencies [31]. Previous studies showed that high stress levels can impair cognitive functions crucial for decision-making [32,33]. Simulation-based MR training enables practical application within environments that mimic the perceptual, motor, and cognitive demands of high-stress situations [34,35].

Real-time measurement of stress in training environments presents unique challenges that differ significantly from traditional posttraining subjective evaluations. Although posttraining assessments often rely on self-reported data, real-time stress assessment requires the integration of physiological monitoring tools that can capture immediate responses to stressors, offering a more objective view of trainees’ stress levels [36,37]. Currently, biosignals such as heart rate (HR), heart rate variability (HRV), electrodermal activity (EDA), blood pressure, respiratory rate, and electroencephalography are commonly used for stress measurement [38].

However, incorporating these biosignal measurements into real-world training environments poses significant challenges. Most research to date has been conducted in controlled lab settings where participants’ movements are limited, minimizing noise in the data [39]. In contrast, training environments that involve high levels of physical activity introduce additional difficulties, as movement can introduce noise and artifacts into biosignal recordings.

Despite the potential of these physiological markers, achieving accuracy and reliability in real-time stress measurements, especially in dynamic training environments, remains a complex task [40]. The challenge lies in the need for sensors that can reliably track these biosignals during movement-intensive scenarios and the development of algorithms capable of processing and interpreting data in real time without introducing latency.

The visualization of physiological signals (ie, HR, HRV, and breathing rate) is a key component in biofeedback applications designed to help individuals understand and manage their own stress levels [41]. Research on biosignal visualization has shown that presenting physiological data in an accessible, user-friendly format can enhance self-awareness, allowing individuals to recognize stress responses and use strategies to mitigate them [42]. However, limited research can be found about visualizing stress data for third-party observers, such as trainers or supervisors, in a way that is informative and actionable. Research on the visualization of physiological data in XR is limited as well and mainly involves investigating its influence on social factors, such as social connections [43]. Further research is needed to develop more sophisticated visualization tools that can convey essential information quickly and discreetly, enhancing the utility of real-time stress monitoring in training contexts.

## Methods

### Overview

**Figure 1.**
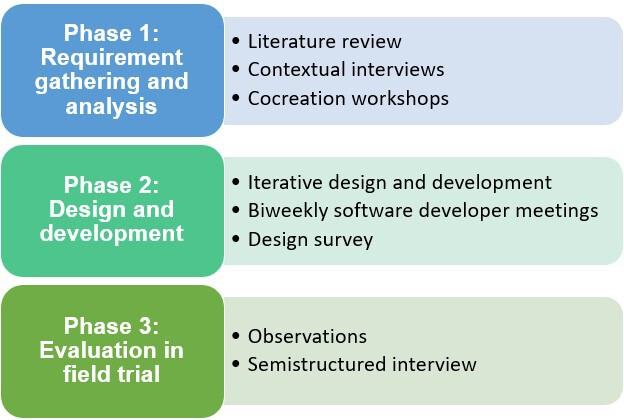
Research protocol: 3 phases and methods.

All studies described in this manuscript are part of the Horizon 2020 project MED1stMR [44]. The goal of the research project was to design an MR simulation training system for MFRs that is based on the needs and preferences of end users. This study focused specifically on enhancing immersive training environments to improve performance and stress measurement, as these were identified as critical areas of concern by the consortium members. The methodology included a structured multiphased research structure with a strong focus on human-centered design methods. The research protocol included a phase for requirements gathering, as well as iterative design and development and evaluation, as shown in Figure 1.

### Recruitment

MFRs and trainers (also referred to as end users) were recruited from the following consortium members to participate in the studies: Servicio Madrileño de Salud, Hellenic Rescue Team, Johanniter Österreich Ausbildung und Forschung Gemeinnützige GmbH, Region Jämtland Härjedalen, University Hospital Heidelberg, and Campus Vesta, as well as external partner Sanitätspolizei Bern.

For the contextual interviews and cocreation workshops, participants were recruited via email and word of mouth between October and December 2021. The online survey was distributed in January 2023, with each organization sending an invitation link and a brief description of the study to their MFRs.

For the field trial, participants were recruited through Johanniter Österreich Ausbildung und Forschung Gemeinnützige GmbH from April to June 2023, using email and word of mouth as the primary recruitment methods.

### Requirements Gathering and Analysis

For the collection of end user requirements, contextual interviews were held with 30 participants (16 female, 14 male) and 41 MFRs (19 female, 22 male) who contributed to cocreation workshops (the interview guide was developed with MFRs and research experts from the consortium and can be found in Multimedia Appendix 1). The goal of the cocreation workshops was to engage end users in the process of scenario design and collect input regarding training goals and features. Workshops included traditional and immersive prototyping as Nguyen et al described in a separate study comparing the 2 techniques [45].

As part of the structured interviews, participants were asked to list the KPIs their organization is using to assess trainees in real-life MCI training, including assessment methods, rating scale (eg, categorical, numerical, checklist), and time of assessment (during training or debriefing). Upon completion of the interviews, all KPIs, along with descriptions, were compiled into an Excel sheet (Multimedia Appendix 2) and categorized by assessment type (eg, safety-related aspects, triage performance, application of triage algorithms, treatment of patients, use of equipment, and communication and coordination). The MoSCoW method [46] was used to prioritize KPIs. This method rates each item as must have (Mo), should have (S), could have (C), or will not have (W). The single point of contact for each end user organization coordinated with their team to complete the ratings. In the next step, the technical development team added information about feasibility and resource requirements for each item in the list to get a better understanding about the scope.

To receive input regarding the stress level indicator specifications, participants were asked about their preferences regarding biosignal recording wearables (eg, chest strap, smartwatches, sticky electrodes on the chest) and biosignals to use for stress classification (eg, electrocardiogram [ECG], EDA).

### Design and Development

In the design phase, an agile development process [47] was used to develop the MR system, including the scenario editor; 2 scenarios; stressors; avatar appearance and wounds; virtual medical tools; performance monitoring; and debriefing tools. The initial requirements collected in phase 1 were transferred to the product backlog and development was divided into manageable, incremental builds, allowing for regular assessment and adaptation. Biweekly software developer meetings were held to foster close collaboration between developers and end users, ensuring that the evolving needs and feedback of trainers and trainees were continually integrated into the development process.

The technical development team was unclear about how the stress level indicator should be visualized and what level of information should be included; therefore, an online survey was conducted via the survey platform LimeSurvey. Mock-ups were created based on best practice (eg, stress indicator of smartwatches) and examples from the literature [48,49] and presented to end users as still images in an online survey (Multimedia Appendix 3). The survey took about 10 minutes to complete and was distributed through the end user organizations of the project. In total, 54 MFR trainers from 8 different MFR organizations across 7 European countries participated.

### Evaluation in Field Trial

#### Overview

Finally, a field trial was conducted to gather feedback from end users and observe their interaction with the newly developed features. In total, 36 MFRs (6 female, 28 male) and 4 trainers (1 female, 3 male) participated in the field trial.

The MR training setup encompasses a 10 × 10 training field, allows for teams of up to 4 trainees, and is equipped with a head-mounted display and trackers placed on the trainees’ hands, feet, and back. To ensure seamless communication, trainees and trainers are provided with headphones, which also facilitate role-playing interactions with dispatchers. The training environment is further enriched with 2 patient simulator manikins [50], which offer a tangible patient interaction experience. Additionally, a biosignal collection device [51] is integrated into the system, capturing real-time ECG and EDA sensor data to monitor trainee physiological responses. The current stress level indicator utilizes a combination of HR and HRV to categorize stress into low, medium, and high levels. This approach is adapted from a previous research project about VR training for law enforcement, as described by Zechner et al [52].

After being introduced to the study and signing the informed consent form, participants filled out a demographic questionnaire and received an introduction to the MR system and the task, which involved performing first triage at 2 different MCI sites (1 scenario each). The training was conducted in teams of 4 with 1 trainer, who also voice-acted the emergency dispatcher and included the following scenarios that were conducted in random order.

#### Scenario 1

Emergency teams in 2 ambulances (2 trainees each) respond to a bus-car collision on a highway with multiple casualties. They arrive at the virtual scene after hearing the emergency call from the dispatcher and receive updates about the accident’s specifics such as weather conditions and potential hazards, including a stray dog and a damaged light pole. The trainees’ tasks involve patient assessment, risk management, and clear communication. The exercise concludes with a detailed situation report to the triage commander.

#### Scenario 2

In a tunnel-based MR training scenario, emergency medical teams from 2 ambulances respond to a multivehicle accident caused by a public transport bus colliding with a car that had a tire explosion. The accident involves at least 3 cars and the bus, with around 15 passengers. As the scenario unfolds, trainees face additional challenges, including locating a missing child and managing potential risks from a gasoline-powered and damaged bus. The exercise concludes with a detailed situation report to the triage commander.

During 7 training sessions, observations took place, including checklists for usage of features, reports on predefined events, time stamps, and open notes, which were later organized into themes. On the fourth day of the field trial, all 4 trainers participated in a semistructured interview (Multimedia Appendix 1).

### Statistical Analysis

For training observations, both quantitative and qualitative analysis were used to analyze data from training observations. Checklists were analyzed by calculating frequencies and percentages for each feature usage and event occurrence to understand their prevalence. Trainer-trainee interactions were recorded through note-taking and later analyzed by identifying and highlighting significant statements or behaviors and these were grouped into themes.

Interview data were transcribed and subjected to thematic analysis [53]. Codes were assigned to significant statements, which were then grouped into themes to identify common patterns and insights regarding the usability and effectiveness of the MR training system. The thematic analysis of qualitative data was conducted using Atlas.ti.

### Ethical Considerations

All studies within the Med1stMR project were approved by the Karl Ruprecht University of Heidelberg (Antrag AZ Beu 2023 1/1, August 15, 2023).

## Results

### Phase 1: End User Requirements

End users expect the technology in MR training to be user-friendly and cost-effective, while improving training outcomes and safety. They desire a system that is intuitive to use and operates smoothly to foster broad acceptance within their organization. They also anticipate that the technology will be more cost-effective than traditional MCI simulation training in the long run. MR training is expected to enhance training outcomes by accommodating more participants, improving the efficiency of training time, enhancing the quality of debriefing with detailed and objective information, and offering the opportunity for trainees to assume different roles in repeated simulations.

Workshop participants identified key aspects of MCI training, including scene safety assessment; internal and external communication; coordination; first and second triage; trauma assessment and treatment; prioritizing for transport; and both prehospital and in-hospital MCI management. Participants underscored the importance of enhancing the quantity and quality of training with regard to scene safety assessment; communication and coordination; and triage procedures, which are currently lacking in MCI training. Triage training often relies on paper patients or role-players, both offering limited realism. Paper patients (paper cards that list a patient’s injuries and vital signs) reduce the complexity of the triaging process. Role-players, despite being able to simulate a victim’s status using make-up and behavioral cues, are inherently limited in representing patient symptoms. MR simulations allow MFRs to develop a deeper understanding of MCI procedures, thus improving preparedness and response efficacy.

Regarding KPIs, during the end user requirement collection phase, we gathered 80 performance metrics, of which 57 where prioritized as high and 56 were selected for live tracking. After consolidation of items that were similar enough, a total of 54 performance metrics (42 high priority and 36 marked for live tracking) remained. With the aim to reduce this number to a realistic and implementable workload, all metrics were categorized into the following KPI groups: 3S (scene, safety, situation); triage performance; patient assessment and treatment; use of equipment; communication and coordination; and team performance.

Trainers emphasized that monitoring KPIs in real time would help them better understand a trainee’s progress and areas of improvement. In a dynamic training environment, capturing metrics such as response time, decision accuracy, status of the patient simulation manikin, and task completion rate can reveal valuable insights about a trainee’s aptitude and readiness for real-world scenarios. Real-time performance monitoring can also facilitate immediate feedback, a process that can significantly enhance the learning progress by highlighting areas of strength and those needing improvement promptly after or even during training sessions.

However, not all performance metrics are equally important in every scenario but rather depend on the specific training objective. To cover a variety of training goals and scenarios, a library feature has been requested for the MR training platform, allowing trainers to select up to 8 KPIs for real-time monitoring.

Furthermore, it has been highlighted that monitoring team performance metrics such as task distribution, communication efficiency, and collaborative decision-making can offer a comprehensive understanding of team dynamics and synergy, in addition to individual KPIs [54].

Furthermore, the workshops with end users underscored a pivotal shift from merely focusing on physical safety to a more holistic approach that encompasses psychological well-being. Although MR training reduces physical risks compared to real-life simulations, the heightened realism and immersive nature can increase trainee stress. Instructors should be able to monitor trainees’ stress levels, ideally based on biosignals, throughout the training exercise.

End users also pointed out the need to identify specific stressors for MCI scenarios. These stressors can range from environmental factors (like poor visibility) to operational challenges (such as resource shortages). One notable stressor is the condition of the victims in the scenario. Their health status, both physical and mental, can impact trainee stress. Therefore, it is essential to detail the victims’ health trajectory throughout the training, including key physiological parameters during critical stages.

The visualization of stress levels was a frequently discussed topic by end users, who considered stress level monitoring as a must-have feature of the platform but were concerned about the feature distracting the instructors from their already-high task load. Showing trainees their own and colleagues’ stress level was dismissed early in the project because it would not resemble a real MCI and this was viewed as critical for privacy reasons.

The following requirements were identified:

- Visibility: Stress levels should be visible only to trainers to maintain trainees’ immersion.
- Accessibility: Stress levels should be continuously visible to trainers without active involvement (eg, mouse clicks).
- Association: It should be easy to link the stress visualization to the respective trainee’s avatar.
- Interpretability: The visualization should be straightforward, avoiding complex numerical displays.
- Distraction: The design should ensure the visualization is noticeable but not distracting.

These insights from the workshops provided valuable guidance for designing MR MCI training that is both effective and mindful of trainee well-being. Following the guidance of experienced trainers, stress level visualization options were designed and tested with end users through an online survey, as reported in the next section.

### Phase 2: Design and Development

#### Overview

The design and development phase was the core phase of this research project and lasted for 14 months. During this time, prototype tryouts had been organized for end users to test the current version of the system, monthly consortium meetings were held to discuss the current status and gather feedback, and biweekly software developer meetings took place online.

KPI implementation was discussed in software developer meetings, leading to further refinement of KPI requirements. Not all KPIs were available in real time, either because they required manual input from trainers or because data transfer and processing could only be done offline. Initial usability testing of the basic prototype, conducted during the design and development phase, revealed that users found it very difficult to monitor more than 10 KPIs simultaneously during training sessions. This finding informed the design adjustments made to ensure the interface remained manageable and focused for trainers during real-time training. From the 54 performance metrics identified by end users, a subset (Table 1) was prioritized for real-time tracking and display, aiding trainers in effectively steering the training sessions.

**Figure 2.**
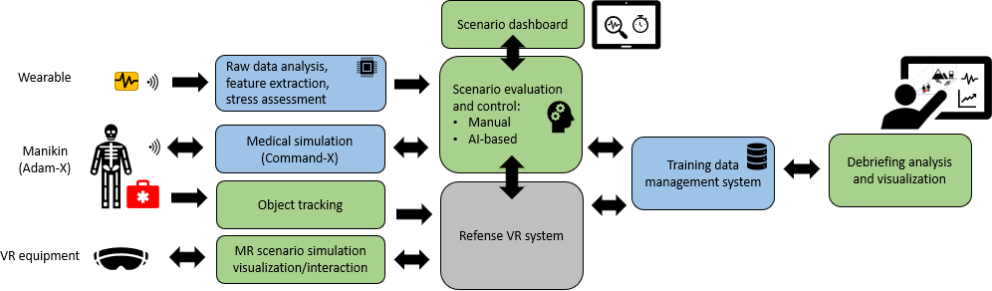
MED1stMR system description. MR: mixed reality; VR: virtual reality.

Real-time stress level monitoring was enabled by wearables and biosignal sensors (Figure 2). After evaluating various wearables and biosignal sensors, end users preferred a chest belt or sticky electrodes, both of which were integrated into the system for further testing (Table 2). Participants suggested useful biodata sources for real-time stress measurement, including HR, breathing rate, brain activity, and galvanic skin response. However, they expressed a preference for following state-of-the-art methods, acknowledging their lack of expertise in this field.

**Table 1.** Summary of key performance indicators mapped to training goals.

Metric	Description	Training goal
Overall timing of scenario	Time taken by trainees to make medical decisions and provide an incident overview	Organize and coordinate work at mass casualty incident efficiently
3S (scene, safety, situation) assessment	Evaluation of scene, safety, and situation; identifying and mitigating environmental risks and hazards	Evaluate and manage the safety and security of the scene
Trainee stress levels	Monitoring the stress levels of trainees during the training to adjust challenge levels	Effective decision-making capabilities in highly stressful situations
Communication skills	Assessing the clarity and precision of communication among trainees	Effective team coordination and information exchange in emergencies
Patient assessment	Evaluating the trainees’ ability to assess patients using the Airway, Breathing, Circulation, Disability, Exposure (ABCDE) approach	Accurate and timely patient care and prioritization
Time taken to triage	Time spent by trainees on triaging each patient	Identifying delays in patient assessment and intervention
Medical task accuracy	Accuracy of medical procedures performed (eg, correct application of a tourniquet)	Performing medical tasks correctly and efficiently

**Table 2.** Summary of requirements for the smart wearables.

Features and needs		Notes
**Free movement and disturbance-free**		
	Wearables should not impede the trainee’s ability to do their job	This requirement is different between the training goals of the training modules “first arriving ambulance,” first triage, and second triage
	Wearables should not be “felt” (eg, tightening of a blood pressure cuff)	To allow immersion in the simulation world and not distract trainees
**Wearable form**		
	Form follows function. Potential wearable forms include a wristband, watch, suit, vest, or shirt.	The participants were open to various forms that the smart wearable could take, whichever would make it easiest to measure the needed biodata streams
			Not too many wearables (ideally 1 integrated wearable)	The trainees do not want to wear ≥4 wearables
	**Measurement**		
	Stress level	Stress was noted as the most important measure
	Situational awareness	Situational awareness was mentioned as a “nice-to-have” measurement

#### Design Preferences of Stress Level Indicator (Online Survey)

Results of the design survey clarified visualization preferences of end users and helped technical development teams implement the various visualizations.

**Figure 3.**
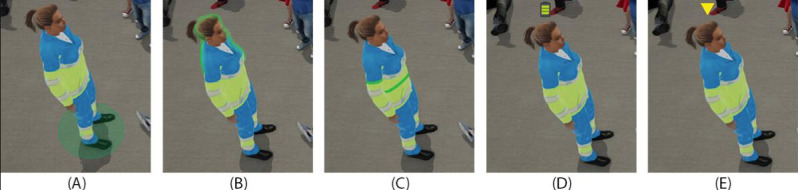
Five examples of real-time stress visualizations presented to end users. A: circle; B: aura; C: belt; D: battery; E: triangle.

Respondents provided feedback on five different real-time stress level indicators in the VR view (Figure 3): (1) circle, (2) aura, (3) belt, (4) battery, and (5) triangle, which were displayed in random order.

The highest overall preference was indicated for the circle, ranked as most preferred by 12 participants (55%), followed by the triangle with 4 participants (18%), icon and aura with 3 participants each (14%), and no participants indicated the belt as the most preferred option.

**Figure 4.**
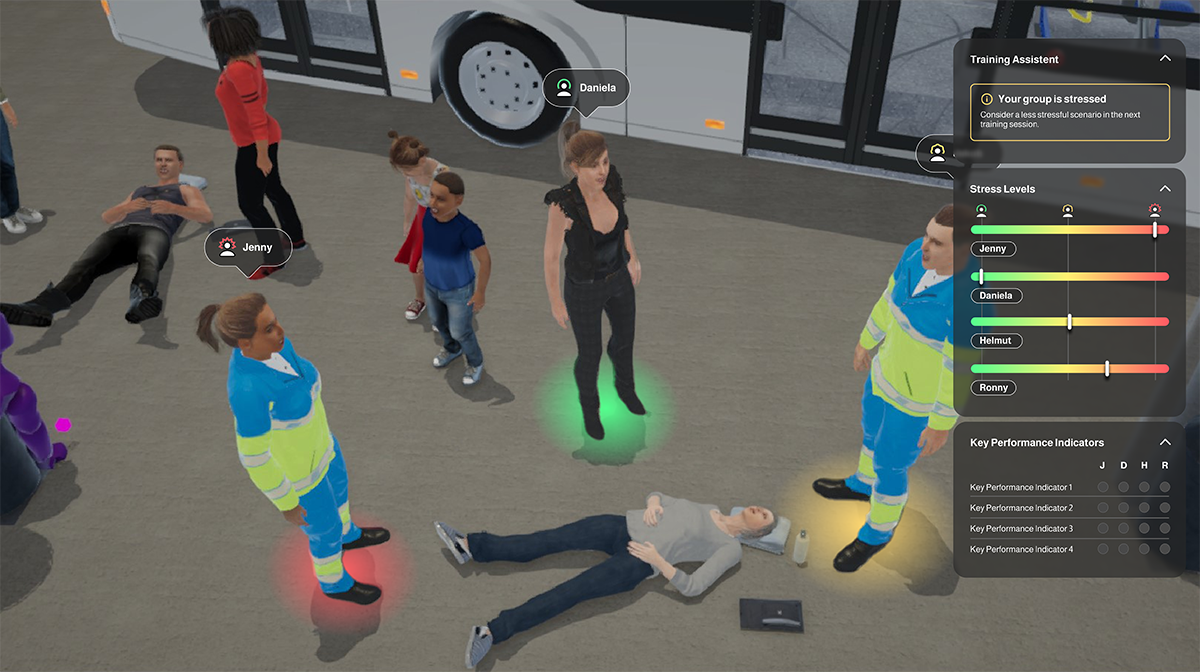
Trainer view of the mixed reality interface (left: view of the virtual environment; right: real-time performance monitoring).

For the side panel (Figure 4), we wanted to know what color scheme trainers preferred to indicate trainee’s current stress levels. The traffic light scheme was considered more appropriate (22 participants, 65%) than the heat map style (12 participants, 35%). Regarding the graph type, the majority of participants found horizontal bars easy to read and interpret (18 participants, 55%) compared to a gauge type (16 participants, 48%) and vertical bars (10 participants, 30%).

Based on the survey results, a stress visualization interface was created (Figure 4).

### Phase 3: Evaluation in Field Trial

**Figure 5.**
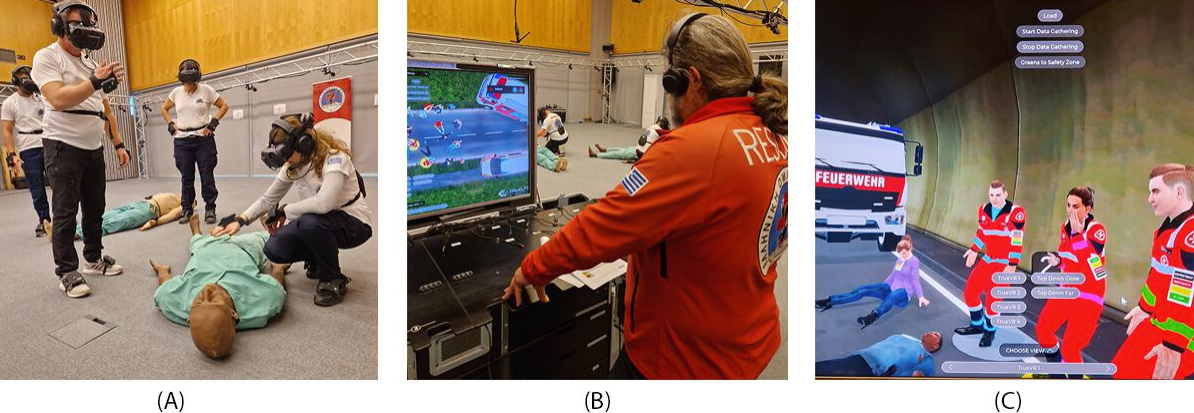
MED1stMR field trial to evaluate features of the mixed reality training platform. A: patient simulator manikin; B: trainer station; C: tunnel scenario in VR view. VR: virtual reality.

During the field trial, training observations of 7 training sessions took place. Each session consisted of 4 trainees who went through an introductory scenario to get familiar with the MR system, followed by 2 training scenarios and a debriefing. On the last day of the field trial, semistructured interviews were conducted with 4 trainers to gather insights on the real-time performance metrics and their utility during MR training (Figure 5).

During the training sessions, our observations revealed a consistent utilization pattern of performance evaluation features by trainers. All trainers routinely accessed the feature that allowed them to verify which triage card was assigned to each patient and if it matched with the recommended card. This feature was particularly popular toward the end of the scenario, ensuring that all patients had been triaged appropriately.

Although minor corrections were occasionally given to trainees between scenarios, comprehensive performance feedback was reserved for the posttraining debriefing sessions. This approach emphasized the importance of performance metrics during debriefings rather than real-time interactions during training. However, the recorded real-time tracking of KPIs was consistently used in every debriefing session. Spanning approximately 20 minutes, these sessions highlighted the KPI tracking feature as an invaluable tool, a sentiment echoed by the trainees.

Each trainer had conducted at least 3 training sessions before the semistructured posttraining interviews.

Trainers identified the following as the most crucial real-time performance metrics in MR training:

- Overall timing of scenario: all medical decisions should be made within 10 minutes (depending on the number of casualties) and trainees should be able to give the dispatcher an overview of how many red, yellow, green, and black patients are at the incident site
- 3S (scene, safety, situation) assessment (ie, identify, communicate, and reduce any potential environmental risks or hazards)
- Trainee stress levels (allows trainers to intervene if trainees appear not challenged enough)
- Communication (clear and precise)
- Patient assessment and triage decision (Airway, Breathing, Circulation, Disability, Exposure approach to assess and treat the patient)
- Time taken to triage (aids in intervention if trainees spend excessive time on a single patient)
- Medical task accuracy (eg, application of a tourniquet)

Although trainers emphasized limiting the instructions given during simulation training, they acknowledged the automated tracking’s value. It provided a rapid overview of the number of patients triaged, the accuracy of triage, and the specific trainee responsible. The “time to triage” metric was particularly beneficial, enabling trainers to prompt trainees (eg, by requesting a status update via dispatcher radio) when they took too long.

All trainers reported frequently consulting the circle under the avatar to check trainees’ stress levels and claimed to have had a good overview at all times throughout the training.

For subsequent updates, trainers expressed a desire for enhanced interactivity options to modulate trainee stress levels. This could be achieved through (1) patient simulators and nonplayer characters with adjustable parameters like pulse and breath rate or (2) the ability to introduce audiovisual stress cues ad hoc. Furthermore, more time in the tutorial scenario was requested, to give trainees the chance to test all features at least once and get familiar with the virtual environment and how to interact with patient avatars. They reported that it was sometimes not clear if a trainee struggled because of the MR technology or their skills and knowledge, which is relevant when evaluating their performance.

## Discussion

### Principal Findings

This research aims to enhance MR training for MFRs by addressing key research questions. First, we identified crucial performance metrics, such as scene safety assessment, communication efficiency, triage accuracy, and patient management, which enabled trainers to effectively engage with and mentor MFR trainees. Second, we integrated a stress level indicator into the MR training platform using real-time biosignal analysis, allowing trainers to monitor trainees’ current stress levels during the training session.

Incorporating a performance monitoring tool emerged as a high-priority request from MFR organizations during the requirement collection phase. The integration of KPIs into MR training provides a structured and measurable approach to evaluating trainee performance and supports trainers in the debriefing process. End users consider this a significant advancement over traditional methods, which often lack objective performance metrics. Literature [27,54] suggests that the inclusion of real-time performance indicators can significantly enhance learning outcomes by providing immediate feedback and enabling targeted interventions. Our study extends this knowledge by demonstrating the practical application of KPIs in MR training, with trainers identifying key metrics such as overall timing, 3S assessment, and communication skills as crucial for effective training. Although most KPIs appeared highly important during the requirements collection phase, field trials led to a refined focus on actionable and timekeeper metrics for display in real time. Other KPIs were deemed more suitable for posttraining debriefings. This shift in priorities underscores the evolving understanding of KPI relevance as trainers gain hands-on experience with the MR platform. Although MR technology offers a seemingly rich array of tracking possibilities, the key lies in selecting and translating the most impactful KPIs to ensure effective engagement and mentoring of MFR trainees.

Both trainers and trainees appreciated features incorporated into the virtual environment that provided immediate feedback, such as the cessation of bleeding only when a tourniquet is correctly applied. Such immediate feedback mechanisms enhance the training experience by providing real-time validation or correction while fostering a more immersive and instructive environment.

The capability to repeat scenarios multiple times in MR training has been highlighted as a significant advantage over traditional real-world simulation training because it allows trainees to refine their skills through practice [55]. Enhancing MR training platforms with real-time performance monitoring further empowers trainers with the ability to adjust scenarios on the fly based on the actual performance of trainees. This dynamic adaptability ensures that training sessions are not only more efficient but also personalized to meet the unique needs and skill levels of individual trainees. Real-time monitoring provides trainers with immediate insights into trainees’ performance, enabling them to tailor scenarios to challenge and develop specific competencies.

The inclusion of stress level monitoring in our MR training platform addresses a critical gap in traditional MCI training methods, which often overlook the psychological well-being of trainees. Previous research has highlighted the impact of stress on decision-making and performance in emergency scenarios [34]. A stress level indicator can be seamlessly integrated into the MR training platform by using biosignals for real-time monitoring through wearable sensors, such as chest belts, sticky electrodes, or watches [41]. The stress level indicator should be visible only to trainers, continuously accessible without active interaction, easily associated with trainees’ avatars, straightforward to interpret, and designed to be noticeable without being distracting. The preferred indicator, as per user feedback, is a simple and easy-to-interpret visual element, like a circle attached to the trainee’s avatar with 3 stress level thresholds (low=green, medium=yellow, high=red).

Displaying real-time stress levels was regarded as a highly valuable and innovative feature of MR simulation training. For trainers, it enables quicker identification of whether a trainee is overwhelmed or underengaged, allowing for immediate intervention, thus maximizing the efficiency of training time. For trainees, this ensures their safety and psychological well-being, while also fostering optimal training environments that are conductive to effective learning and skill development.

In this research, a relatively simple stress model was used [52], calculating a combination of HR and HRV and categorizing the results into 3 groups: low, medium, and high. Our focus was on visualization and interface integration, as this was a major concern of our end users and the current literature showed a noticeable gap regarding the detailed exploration of these aspects. However, more comprehensive and robust models for stress assessment are documented in existing research [38,56]. These models often adopt a multimodal approach, incorporating measurements like ECG-related features and EDA [36].

Regarding stress level visualization, while no specific research was found on techniques for conveying stress levels to third-party observers (eg, trainers) in MR, our approach drew inspiration from biofeedback [57], biosignal visualization methods [58], and social biofeedback systems outside the MR domain [43].

### Strengths and Limitations

A key strength of this research lies in its user-centered design process and the evaluation of MR training in real-life training settings. Engaging end users and trainers throughout the project allowed us to tailor the system to the specific needs and challenges faced by MFRs. By conducting field trials and workshops with professionals from various European countries, the study provided actionable insights into how MR training can enhance skill development. Additionally, the iterative feedback from trainers was instrumental in refining performance metrics and the user interface, ensuring the training system’s practicality and relevance to real-world scenarios.

However, this research also has several limitations. First, there may be a selection bias since the end users and trainers involved were volunteers rather than randomly selected participants. Although we attempted to mitigate this by recruiting a diverse group from multiple European countries, the sample may still not fully represent the broader population of MFRs. This limitation may influence the generalizability of our findings. Future research should consider a randomized participant selection process to improve representativeness and enhance the validity of the results.

Second, the relatively small sample size of trainers and trainees in the field trial limits the generalizability of the findings. Although the study provided valuable insights, the experiences observed may not reflect those of a larger, more diverse group of MFRs. To address this limitation, future studies should aim to include a larger sample size, which would offer more robust and widely applicable insights.

Third, integrating MR training into existing curricula requires further investigation to understand how this technology can be effectively adopted within current emergency response frameworks. Although this study focused on developing and refining the MR training system, future research should explore best practices for curriculum integration and acceptance within emergency response training programs.

Last, the current assumption that including performance metrics and stress level monitoring in MR training will improve learning outcomes and transfer to real-world MCIs is mainly based on existing literature and the experiences of trainers and subject matter experts. This could have influenced the study’s approach and conclusions. To evaluate the sustained impact of these interventions, a longitudinal study is recommended. Tracking trainees over time would provide a more comprehensive understanding of the long-term benefits and applicability of MR training in real-world situations.

Despite these limitations, the study lays a solid foundation for future research by providing initial insights into the effectiveness of MR training for MFRs and identifying areas for further development.

### Future Directions

Future studies should address these limitations by including a more diverse participant pool, a larger sample size of trainers, and a comprehensive evaluation of curriculum integration strategies. Restricted by the limited resources of this research project, only a small selection of KPI analysis and display was automated; in future work, more sophisticated and automated methods for tracking and evaluating KPIs should be investigated. This includes the development of digital checklists and enhanced data integration systems to facilitate continuous training progress and provide trainers with more robust tools for assessment.

ML algorithms have the potential to significantly improve data processing and pattern recognition in this context. Supervised learning models, such as linear regression, decision trees, and support vector machines, can be trained on historical data to predict KPIs or stress levels [59]. Once trained, these models can provide statistical insights for debriefings or offer direct feedback to trainees based on their performance relative to predicted KPIs.

Unsupervised learning models, such as clustering algorithms like k-means or hierarchical clustering, can be used to group similar performance data together. This can help identify common characteristics of high-performing trainees, which can then be used to better train other trainees [60]. To provide real-time feedback to trainees, reinforcement learning can be used. The model learns to make decisions based on the reward (or penalty) it receives for its actions, which can be tied to the KPIs. For example, a reinforcement learning model could be used in a training simulation to provide feedback to trainees based on their actions [61].

Deep learning models can be used to analyze more complex patterns in KPI data. For example, recurrent neural networks are great tools to analyze time-series data, while convolutional neural networks can analyze image or video-based KPI data, potentially supporting video analysis during the debriefing of the training [62]. Current challenges in automating the analysis of qualitative data, such as communication and team performance, could be overcome in the future by using natural language processing models, which can identify key themes and sentiments in speech-to-text–based data [63]. In our current research, we are investigating the feasibility of the abovementioned machine learning models to test their application in MR training and will report results in future research.

An example, showcasing the power of reinforcement learning to adapt virtual scenarios based on trainee performance, has been provided by the research group developing Unity’s Machine Learning Agents toolkit [64]. This open-source tool was originally developed to create game-based training environments for training intelligent agents. However, in the context of our research, it could potentially be used to develop a smart trainer assistant that adapts virtual scenarios based on continuous trainee performance analysis.

Furthermore, real-time training analysis opens up new possibilities for scenario adaptation and the development of more personalized training guidelines. Future work will explore these avenues and focus on the development of smart scenario control features supported by advanced machine learning methods.

### Conclusions

In conclusion, this research addresses important gaps in immersive simulation training for MFRs by integrating real-time performance metrics and stress level indicators into MR systems. This innovative approach enhances training by providing objective, actionable insights for both trainers and trainees, emphasizing the crucial role of performance evaluation and stress management in emergency response scenarios. Additionally, the study presents insights into user preferences for stress visualization, highlighting the need for intuitive and user-friendly interfaces. The human-centered design methodology ensures these enhancements align closely with end user requirements. The methodologies and design considerations outlined in this work can serve as a framework for developing interactive and immersive training environments across various high-stress fields, contributing to more effective, efficient, and user-focused training solutions.

## Supplementary material

10.2196/57655 Multimedia Appendix 1 Interview guide.

10.2196/57655 Multimedia Appendix 2 Requirements and key performance indicators.

10.2196/57655 Multimedia Appendix 3 MED1stMR survey.

## Abbreviations

ECG: electrocardiogram
EDA: electrodermal activity
HR: heart rate
HRV: heart rate variability
KPI: key performance indicator
MCI: mass casualty incident
MFR: medical first responder
MR: mixed reality
VR: virtual reality
XR: extended reality
