# Supplementary material for: Enhancing Mixed Reality Simulation Training Technology With Real-Time Performance Visualization: Mixed Methods Study With Medical First Responders
Source: JMIR XR Spat Comput. 2024 Dec 24;1:e57655. doi: 10.2196/57655 (PMC13202515; doi:10.2196/57655)
Supplement: Multimedia Appendix 1 [file xr-v1-e57655-s001.docx]

# Appendix A

# Part I: Interview Guide - Requirements

## Key Performance Indicators

With the two scenarios developed in mind, which KPIs does your organization use to assess trainees in such training exercises:

- Include what is assessed / description
- Assessment method
- Rating scales used
- Time of assessment

| *Input text here:* |
| --- |

## Stress Level Indicator

Trainees’ stress levels should be displayed for trainers during the training session.

What bio-signal (e.g. hear rate, breath rate, etc) would you use for stress level classification?

| *Input text here* |
| --- |

These bio-signals need to be recorded via a wearable sensor trainee’s wear throughout the training. What are the most important requirements you have for this?

Are there any preferred devices or body locations?

Are there any no-go placements for devices?

| *Input text here* |
| --- |

# Part II: Field Trial Interview Guide

## Overall Usability

After testing the system, do you feel this MR system is a useful addition to current training methods?

Elaborate on training goals, comparison to other systems and comparison to real-life MCI training:

| *Input text here* |
| --- |

## Key Performance Indicators / Dashboard

Now that you have conducted several training sessions as a trainer, which KPIs were most relevant during the two scenarios? We will cover both (a) during the training (real-time) and (b) debriefing.

What are the most important performance metrics you want to have displayed in real time

during the MR training?

| *Input text here* |
| --- |

What are the most important performance metrics you want to have displayed in the debriefing session?

| *Input text here* |
| --- |

Please discuss the visual aspect of the KPI widget on the Real-time Performance Indicator panel. What did you like? Where do you see possibilities for improvement?

How easy is it to read during training?

How important is this feature for your training style?

| *Input text here* |
| --- |

## Stress Level Indicators / Dashboard

How frequently did you use the stress level indicator (circle below trainee avatar) during each training session?

1-3 times 4-6 times not at all

How frequently did you use the stress level widget in the dashboard (right side of trainer interface) during each training session?

1-3 times 4-6 times not at all

How well do you think the Stress Level Indicator works in terms of accurately displaying the trainees current stress level?

not at all accurate somewhat accurate accurate

Is the categorization (low, medium, high) nuanced enough?

Please elaborate on your trainer experience and feedback you received from trainees:

| *Input text here* |
| --- |

## Future features

What other features, related to real-time performance evaluation and stress, would you like to

see in a future update?

| *Input text here* |
| --- |

Would you trust a training assistant that is based on artificial intelligence, if it provided suggestions on (a) how to improve scenarios and (b) how to increase trainee’s performance? Please

briefly discuss your decision.

| *Input text here* |
| --- |

Other feedback:
