# Supplementary material for: Enhancing Mixed Reality Simulation Training Technology With Real-Time Performance Visualization: Mixed Methods Study With Medical First Responders
Source: JMIR XR Spat Comput. 2024 Dec 24;1:e57655. doi: 10.2196/57655 (PMC13202515; doi:10.2196/57655)
Supplement: Multimedia Appendix 3 [file xr-v1-e57655-s003.pdf]

Design elements for Stress Level Visualization

There are 12 questions in this survey.

Demographics

Which Organization do you work for?

*

Please write your answer here:

What is your current role within your organization?

Please write your answer here:

How many years of professional experience do you have as a trainer for medical first responders?

*

Only numbers may be entered in this field.

Please write your answer here:

How much previous experience do you have with virtual reality?
(Could also be experience outside of work. For example gaming.) *

Choose one of the following answers
Please choose **only one** of the following:

- none
- tried once or twice
- tried several times
- using it less than once per month
- using it once per month
- using it more than once per month

Design Elements: Live VR View

For the next part we would like you to imagine yourself conducting a virtual simulation training with the new Med1stMR VR training platform. The training involves a highly stressful mass casualty incident scene and you are required to monitor trainees stress levels.

In the following part we will present you with a variety of visualization options for this stress level and would like your feedback.
The interface will consist of a (a) "Live VR View" and (b) "Stress assessment panel" (see picture below). In the "Live VR View" you are supposed to be able to get a quick overview of the stress status of your team but not distract you from the training exercise.
The side panel is supposed to give you a bit more insights, if necessary, and can also be used for de-briefing the exercise.

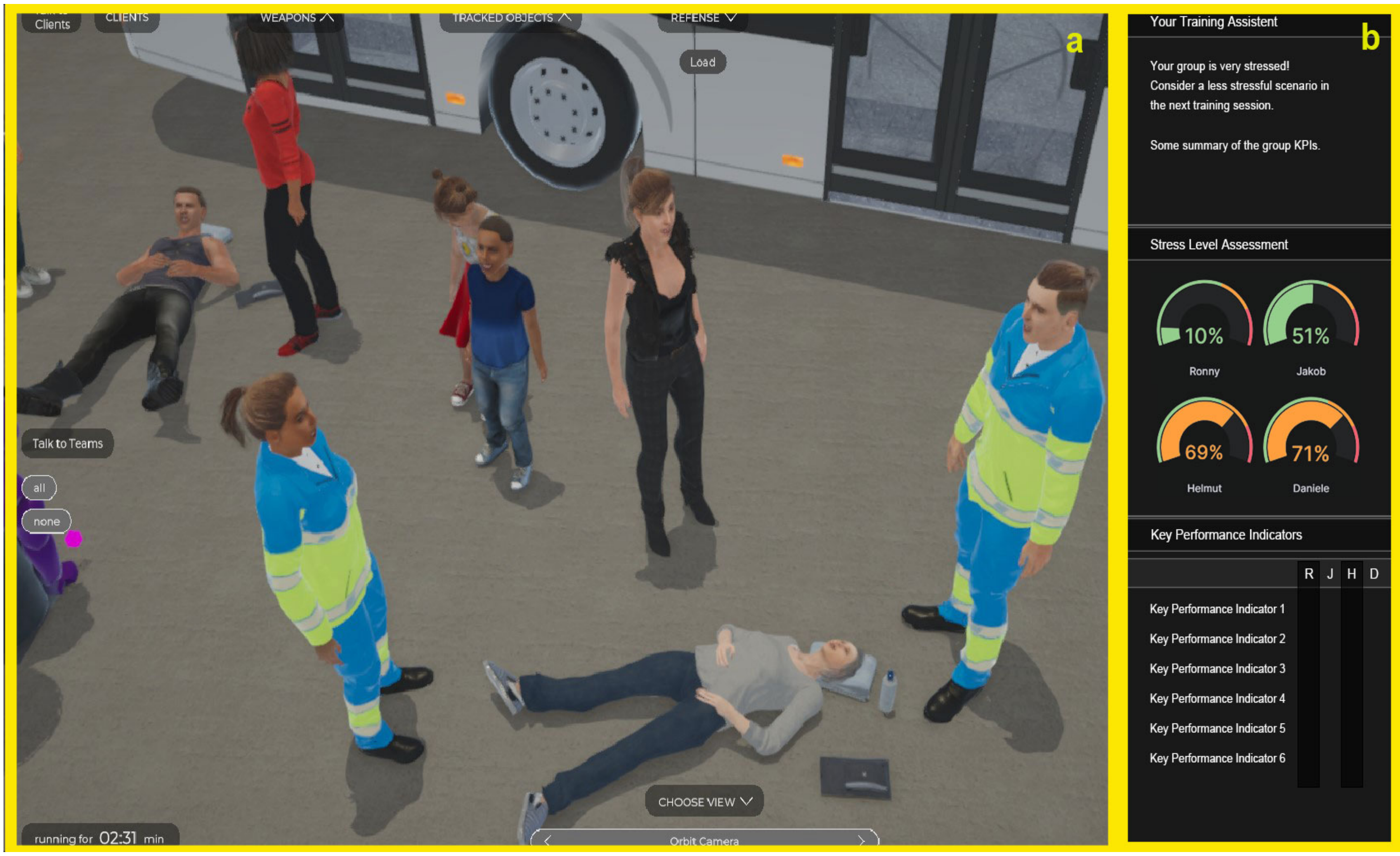

In the following questions we would like to hear your opinion on how to display a stress level indicator in the Live VR View. We have used the following colors to indicate the level of stress (green = normal, red = high)

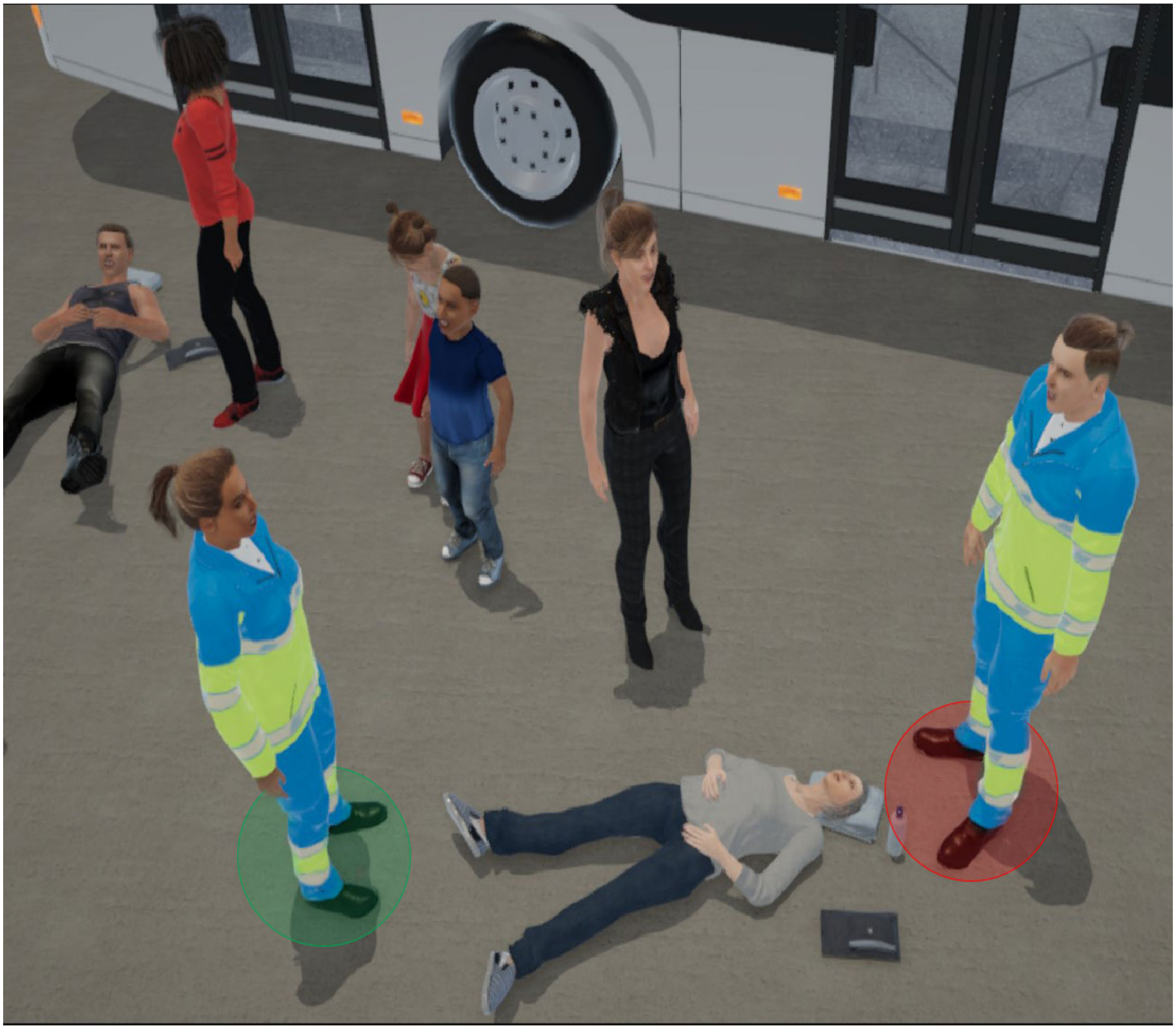

In this scene ... (please select an answer for all 4 questions:) *

Please choose the appropriate response for each item:

	Strongly disagree	Disagree	Neutral	Agree	Strongly agree
**I find it easy to read and interpret the trainee's level of stress**	○	○	○	○	○
**I find the stress level indicator distracting**	○	○	○	○	○

	Strongly disagree	Disagree	Neutral	Agree	Strongly agree
I find the stress level indicator to subtle	◯	◯	◯	◯	◯
I find the stress level indicator useful in a XR simulation training	◯	◯	◯	◯	◯

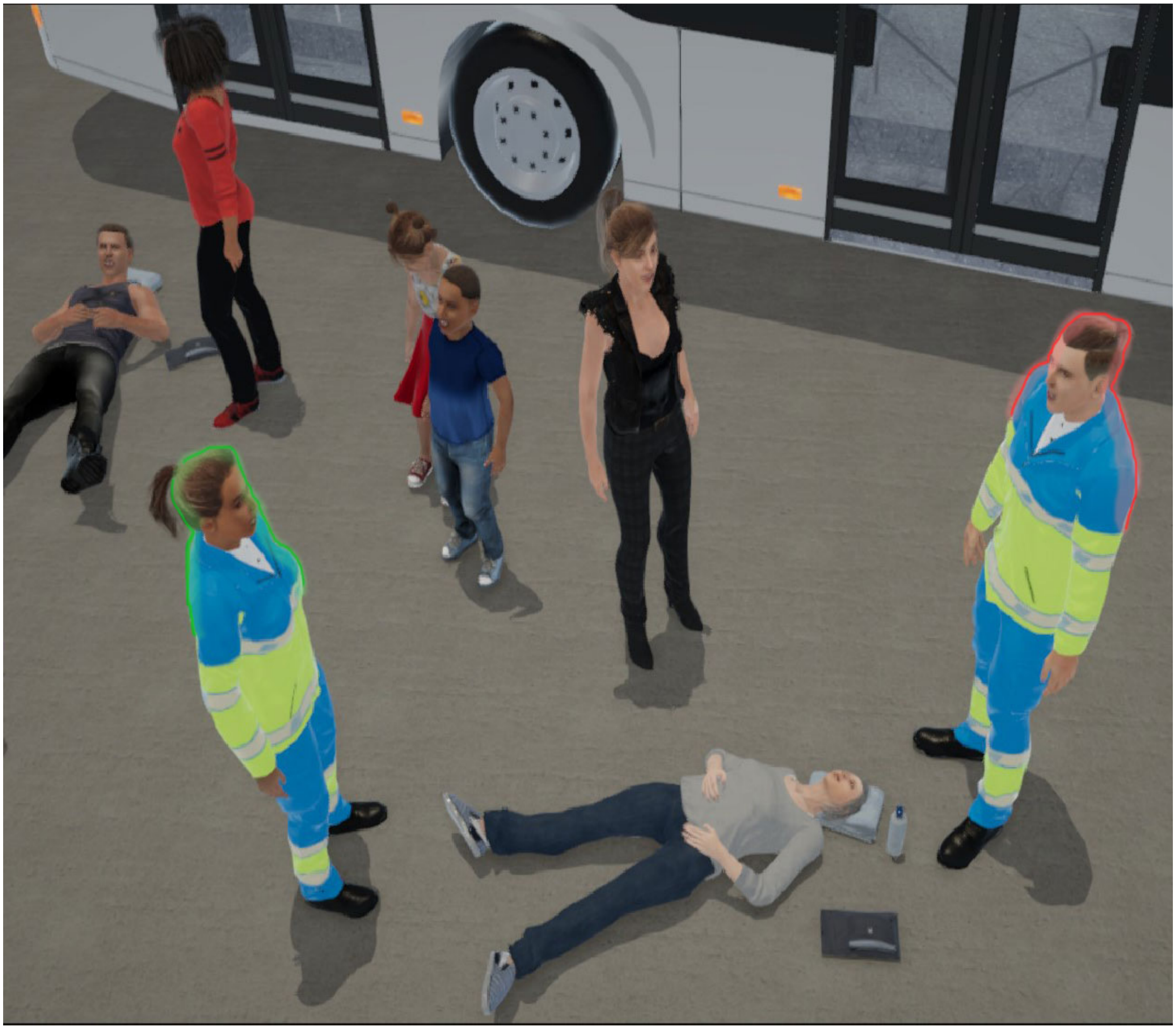

In this scene ... (please select an answer for all 4 questions:)
*

Please choose the appropriate response for each item:

	Strongly disagree	Disagree	Neutral	Agree	Strongly agree
**I find it easy to read and interpret the trainee's level of stress**	◯	◯	◯	◯	◯
**I find the stress level indicator distracting**	◯	◯	◯	◯	◯

	Strongly disagree	Disagree	Neutral	Agree	Strongly agree
**I find the stress level indicator to subtle**	◯	◯	◯	◯	◯
**I find the stress level indicator useful in a XR simulation training**	◯	◯	◯	◯	◯

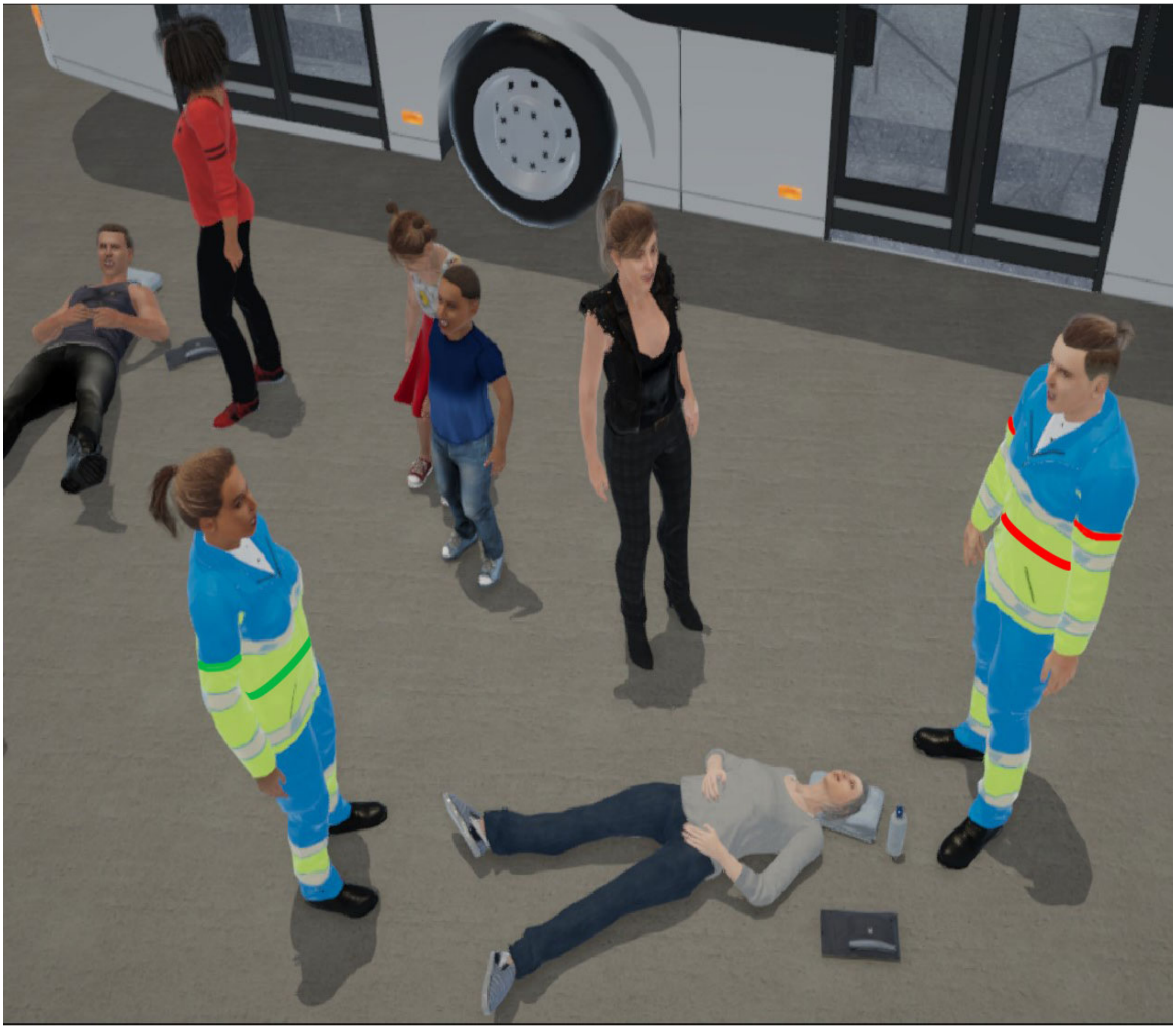

In this scene ... (please select an answer for all 4 questions:) *

Please choose the appropriate response for each item:

	Strongly disagree	Disagree	Neutral	Agree	Strongly agree
**I find it easy to read and interpret the trainee's level of stress**	◯	◯	◯	◯	◯
**I find the stress level indicator distracting**	◯	◯	◯	◯	◯

	Strongly disagree	Disagree	Neutral	Agree	Strongly agree
**I find the stress level indicator to subtle**	◯	◯	◯	◯	◯
**I find the stress level indicator useful in a XR simulation training**	◯	◯	◯	◯	◯

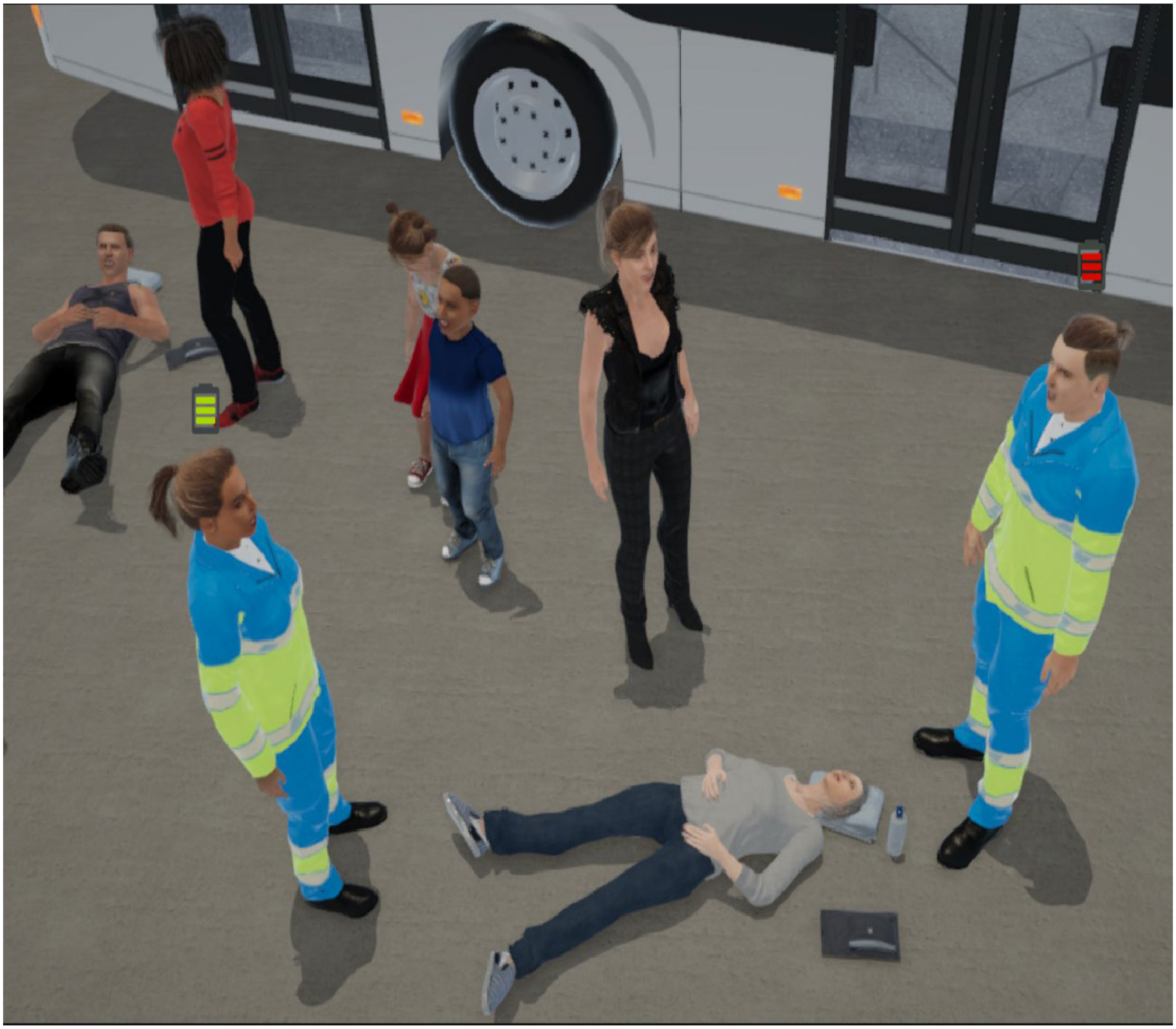

In this scene ... (please select an answer for all 4 questions:) *

Please choose the appropriate response for each item:

	Strongly disagree	Disagree	Neutral	Agree	Strongly agree
**I find it easy to read and interpret the trainee's level of stress**	◯	◯	◯	◯	◯
**I find the stress level indicator distracting**	◯	◯	◯	◯	◯

	Strongly disagree	Disagree	Neutral	Agree	Strongly agree
I find the stress level indicator to subtle	○	○	○	○	○
I find the stress level indicator useful in a XR simulation training	○	○	○	○	○

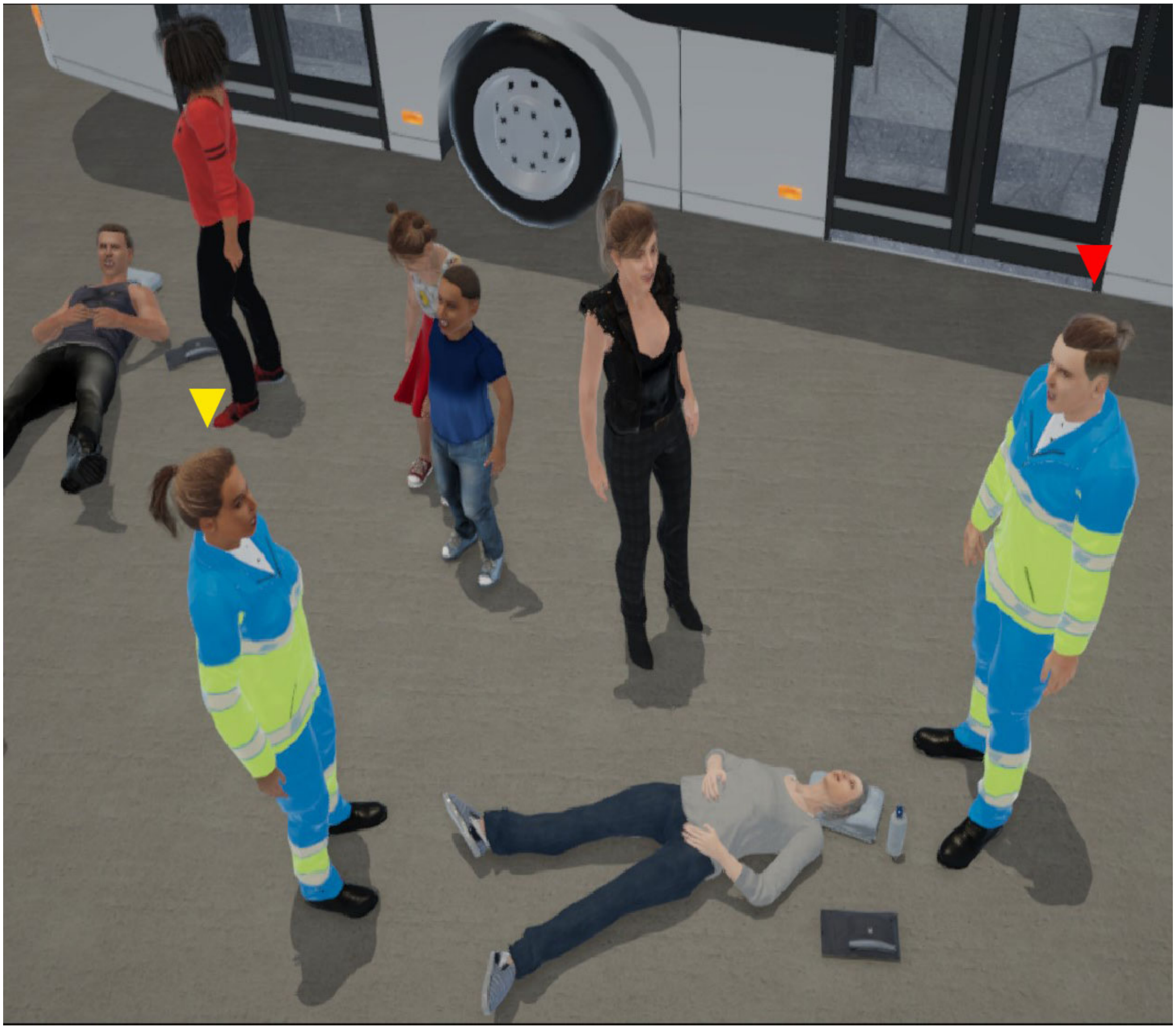

In this scene ... (please select an answer for all 4 questions:) *

Please choose the appropriate response for each item:

	Strongly disagree	Disagree	Neutral	Agree	Strongly agree
**I find it easy to read and interpret the trainee's level of stress**	◯	◯	◯	◯	◯
**I find the stress level indicator distracting**	◯	◯	◯	◯	◯

	Strongly disagree	Disagree	Neutral	Agree	Strongly agree
**I find the stress level indicator to subtle**	◯	◯	◯	◯	◯
**I find the stress level indicator useful in a XR simulation training**	◯	◯	◯	◯	◯

And now please rank all options in order of your preference.

Please number each box in order of preference from 1 to 5

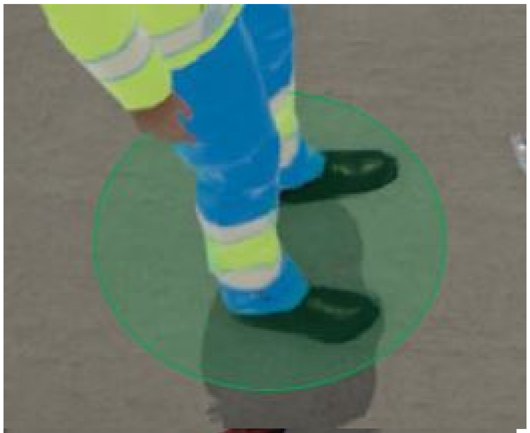

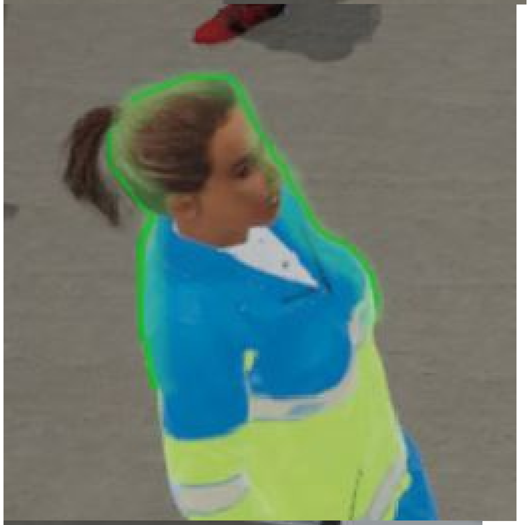

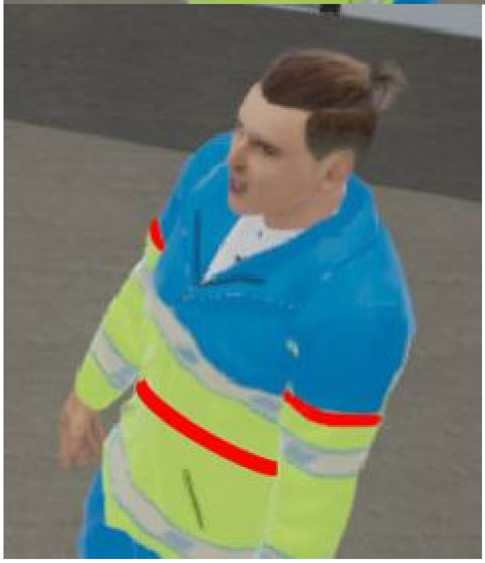

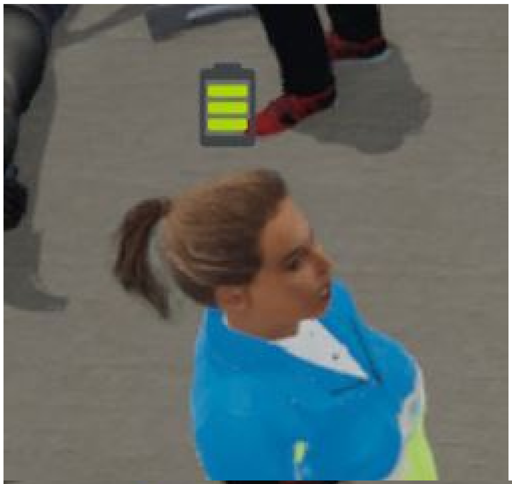

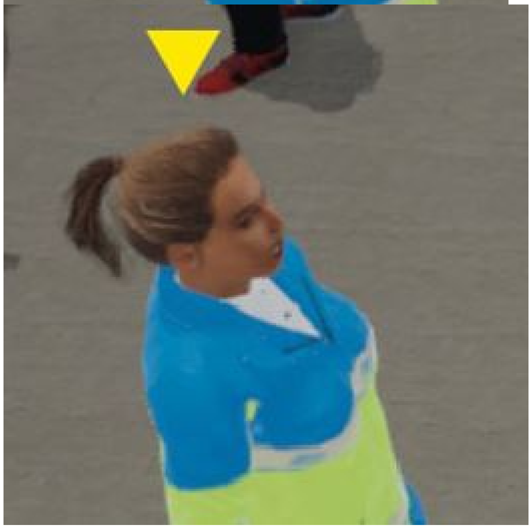

Design Elements: Stress Assessment panel

In the next questions we would like to hear your opinion on features of the side panel.

Which color scheme do you find more appropriate to indicate trainee's stress levels?

(the optimal stress levels for trainees would be somewhere between 40% and 60%). Below that they are not challenged enough, above they might be overwhelmed.

*

Please choose all that apply and provide a comment:

- [ ] 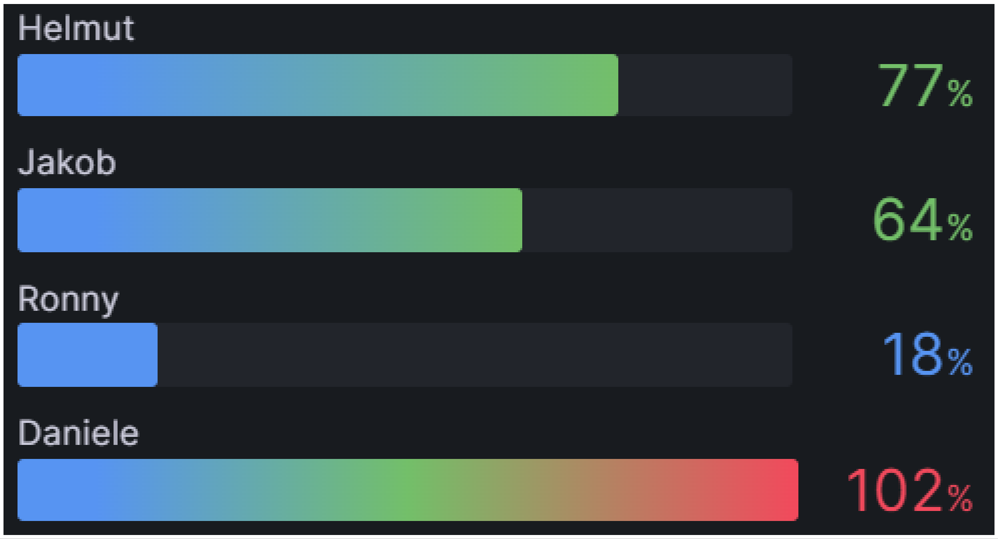

- [ ] 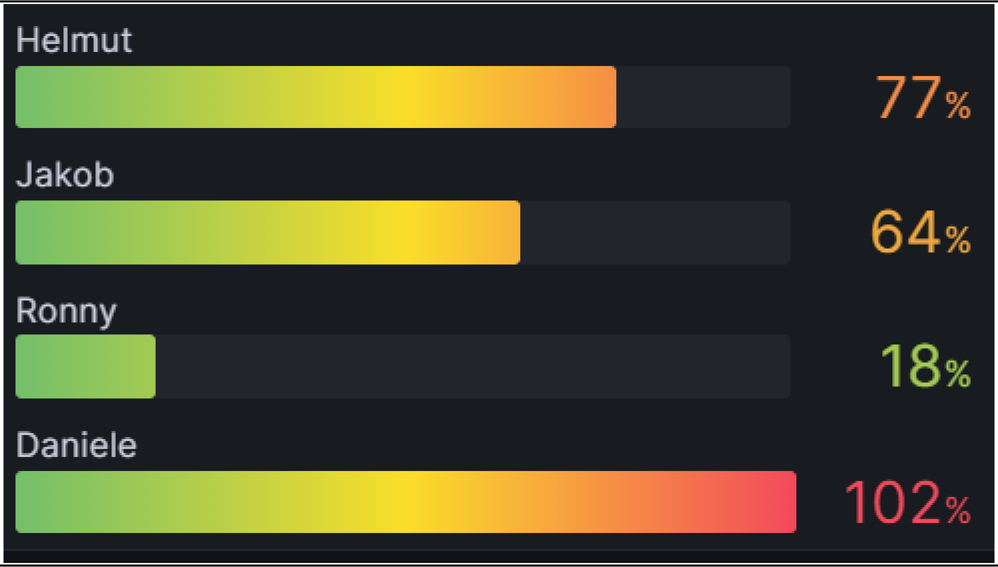

How easy do you consider the following stress level indicators to read and interpret during a live training session? (1 = very easy, 5 = very difficul)

*

Please choose the appropriate response for each item:

	1	2	3	4	5
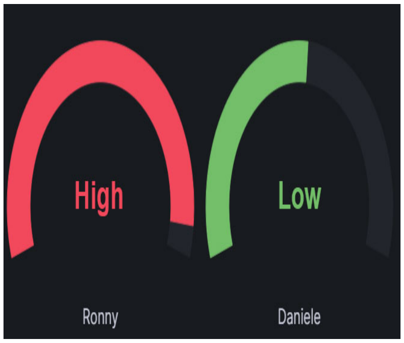	◯	◯	◯	◯	◯
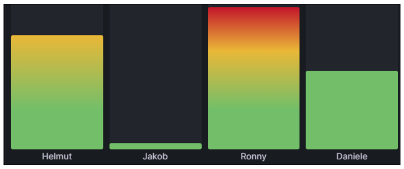	◯	◯	◯	◯	◯
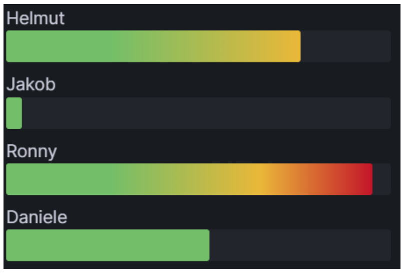	◯	◯	◯	◯	◯

Submit your survey.
Thank you for completing this survey.
